# *Lactobacillus* and *Bacillus* Improve Egg Production in Zhedong White Geese via Gut Microbiota–Metabolite–Endocrine Axis Modulation

**DOI:** 10.3390/vetsci13050479

**Published:** 2026-05-15

**Authors:** Ruilong Song, Biao Wang, Wan Zhang, Xiao Zhou, Shuyan Rui, Qi Wang, Hehuan Li, Xishuai Tong, Hui Zou, Yonggang Ma, Shufang Chen, Zongping Liu

**Affiliations:** 1 College of Veterinary Medicine, Yangzhou University, Yangzhou 225009, China; rlsong@yzu.edu.cn (R.S.); 2 Jiangsu Co-Innovation Center for Prevention and Control of Important Animal Infectious Diseases and Zoonoses, Yangzhou 225009, China; 3 Livestock and Poultry Research Institute, Ningbo Academy of Agriculture Sciences, Ningbo 315000, China

**Keywords:** *Lactobacillus*, *Bacillus*, Zhedong white geese, gut microbiota, metabolite

## Abstract

Enhancing egg production in geese without antibiotics remains a challenge in poultry science. This study compared the effects of *Lactobacillus* (LAB) and *Bacillus* (BAC) probiotics on laying performance, gut microbiota, and serum metabolism in Zhedong White geese. Birds were fed a control diet or diets supplemented with LAB or BAC. Egg production and quality were monitored throughout the trial. Serum metabolomics and fecal 16S rRNA sequencing were integrated with KEGG enrichment and correlation analyses to uncover functional mechanisms. Both probiotics improved laying performance and egg quality. Total egg production of the LAB group was 8.5% higher than that of the BAC group (*p* < 0.05). The LAB group’s advantage in egg production was consistent with its stronger activation of the steroid hormone biosynthesis pathway (elevated serum corticosterone and tetrahydrocorticosterone indicated an overall enhancement of steroidogenic flux). Simultaneously, the LAB group exhibited a more efficient conversion of L-phenylalanine to catecholamine precursors, which drove activation of the neuroendocrine reproductive axis. The BAC group showed more significant changes in nitrogen and energy metabolism pathways and a more pronounced expansion of energy-harvesting Firmicutes. These findings reveal two strain-specific regulatory pathways: LAB functions through the “aromatic amino acid–neuroendocrine–steroid hormone axis,” while BAC relies on the “gut microbiota–energy metabolism” pathway, with direct implications for the precise application of probiotics under antibiotic-free farming conditions.

## Simple Summary

Goose eggs and goose meat are highly favored by consumers for their unique nutritional value. However, improving goose egg production without the use of antibiotics remains a significant challenge in the poultry industry. This study used Zhedong White Geese (an important commercial breed in China) to investigate the effects of two commonly used beneficial feed microorganisms, lactic acid bacteria (e.g., *Lactobacillus acidophilus*) and spore-forming bacteria (e.g., *Bacillus subtilis*), on egg production performance, egg quality, and the intestinal microbiota. A total of 6000 Zhedong White Geese were selected for the experiment and randomly divided into three groups: the control group was fed only the basal diet, while each of the other two groups received the basal diet supplemented with one of the two microbial preparations. The entire feeding period lasted 8 months. The results showed that, compared with the control group, both supplements significantly improved total egg production, eggshell thickness, and egg white quality. Specifically, the total egg production in the *Lactobacillus* group was 8.5% higher than that in the *Bacillus* group. Further analysis revealed that *Lactobacillus* primarily exerted its effects by activating the body’s hormone synthesis system and promoting the conversion of an amino acid called phenylalanine into signaling molecules that stimulate the reproductive cycle, whereas *Bacillus* primarily acted by enhancing gut microbial diversity and improving the body’s utilization of dietary energy. This study elucidated the mechanisms by which different probiotics improve egg-laying performance through distinct biological pathways, providing a scientific basis for farmers when selecting microbial preparations. The findings are of practical value for promoting antibiotic-free, sustainable goose farming, as well as for food safety and environmental protection.

## 1. Introduction

In response to growing global consumer demand for high-protein, low-fat health foods, goose meat and eggs have attracted increasing interest due to their unique nutritional profile [1]. However, the expansion of the goose industry faces several challenges. Intensive farming practices necessitate improvements in both production efficiency and product quality. Moreover, as waterfowl, geese have specific husbandry requirements, particularly high sensitivity to feed efficiency, intestinal health, and reproductive performance. Consequently, nutritional regulation is now regarded as a critical factor in modern goose production [2].

Antibiotics have historically been used in livestock and poultry production to promote growth and prevent disease. Since the 1950s, antibiotic feed additives have substantially improved animal productivity, facilitating industry growth. However, prolonged antibiotic overuse has resulted in serious public and environmental health concerns, including antimicrobial resistance, drug residues, and ecological pollution [3,4]. These issues underscore the need to eliminate antibiotic use in animal production and identify safe, effective, and sustainable alternatives [5].

Among these alternatives, probiotics are regarded as promising due to their ability to modulate gut microbiota, enhance immune function, and improve nutrient utilization [6]. *Lactobacillus* and *Bacillus* spp. have been widely investigated in livestock and poultry. *Lactobacillus*, a common probiotic, inhibits pathogens and improves nutrient absorption via intestinal colonization and the secretion of organic acids and enzymes [7]. It has been shown to enhance growth performance and immunity in broilers. *Bacillus* species, known for their enzyme production and thermal tolerance, enhance nutrient digestion and have been shown to improve productivity in ruminants and dairy cattle [8], as well as laying performance, egg quality, and gut microbiota composition in poultry [9,10]. Despite their widespread application in poultry, the effects of these probiotics on geese, particularly on reproductive performance and gut microbiota, remain poorly understood [11].

A systematic evaluation of the two probiotics is thus necessary to clarify their mechanistic pathways and effectiveness in geese. Critical questions remain: Do these probiotics operate primarily through endocrine modulation, metabolic reprogramming, or microbial composition shifts? How do these mechanisms interact to influence laying performance and egg quality? Answering these questions will not only enable more rational probiotic application in goose farming but will also advance general understanding of microbiome-targeted nutrition in poultry.

To address these issues, this study compared the effects of dietary supplementation with lactic acid bacteria and Bacillus on laying performance, gut microbiota composition, and circulating metabolic profiles of Zhedong White geese. A combined approach of fecal 16S rRNA amplicon sequencing and untargeted serum metabolomics was employed to characterize the strain-specific regulatory mechanisms of each probiotic strain. These findings could deepen our theoretical understanding of the functional mechanisms of probiotics in waterfowl and provide a reference for the rational design of antibiotic-free feeding strategies in sustainable goose production.

## 2. Materials and Methods

### 2.1. Animals and Experimental Design

The Zhedong White geese used in the experiment were sourced from a goose breeding company in Xiangshan. Based on body weight uniformity and health status, a total of 6000 Zhedong White breeder geese (19–20 months of age) were selected and randomly divided into three groups: control group (Con), *Lactobacillus* group (LAB), and *Bacillus* group (BAC), with 2000 geese per group.

The three groups of geese were reared separately in different sections and had ad libitum access to a mixed diet consisting of concentrate, roughage, and green forage during the experiment. The LAB group received 150 g of lactic acid bacteria preparation per ton of feed, with the main active ingredient being *Lactobacillus* acidophilus (viable count ≥1 × 10^8^ CFU/g; lactic acid content 9–11%; moisture ≤ 5%). The BAC group received 500 g of *Bacillus* preparation per ton of feed, containing *Bacillus* *subtilis* spores (viable count ≥5 × 10^10^ CFU/g; moisture ≤ 10%). Both probiotic preparations were commercial feed-grade products provided by Lida Veterinary Medicine Co., Ltd. (Hangzhou, China). The ingredient composition and nutrient levels of the basal diet are shown in Table 1.

### 2.2. Production Performance Assessment

Laying performance was monitored continuously from October 2022 to May 2023, covering an 8-month production cycle. Eggs were collected and counted daily by trained farm staff, with monthly totals accumulated to derive total egg production per group. To assess fertility, collected eggs were incubated and candled on days 6 and 12 to identify and remove infertile eggs, early embryonic deaths, and late embryonic deaths. Fertilization rate was calculated as:


Fertilization Rate (%) = [(Total eggs incubated − Infertile eggs − Early-dead embryos − Late-dead embryos)/Total eggs incubated] × 100%.

For egg quality assessment, 12 eggs per group were randomly sampled in December. Egg weight was determined on an analytical balance, shell thickness with a vernier caliper, and albumen height with a dedicated albumen height gauge. Haugh unit (HU) was derived using the formula HU = 100 × log(H − 1.7 W^0.37 + 7.6), where H is albumen height (mm) and W is egg weight (g).

### 2.3. Serum Collection and Untargeted Metabolomics

At the peak of egg production (December), 10 geese were randomly selected from each group and blood was collected following a 12 h fast. A 5 mL whole blood sample was collected from each goose via brachial venipuncture into a vacuum blood collection tube. After being allowed to clot at 4 °C for 30–60 min, the sample was centrifuged at 4000 rpm for 5 min at 4 °C. The resulting serum was stored at −80 °C for later analysis.

The serum protein precipitation procedure was as follows: 100 μL of serum was mixed with 400 μL of pre-chilled 80% methanol, vortexed for 30 s, and then incubated on ice for 5 min. After centrifugation at 15,000× *g* for 20 min at 4 °C, the supernatant was collected and diluted with water to a final methanol concentration of 53%, followed by another centrifugation under the same conditions. The resulting clear supernatant was used for UPLC-MS/MS analysis.

Chromatographic separation was performed on a Hypersil Gold C18 column (column temperature: 40 °C; flow rate: 0.2 mL/min). In positive ion mode, mobile phase A was 0.1% formic acid in water and mobile phase B was methanol. In negative ion mode, mobile phase A was 5 mM ammonium acetate buffer (pH 9.0) and mobile phase B was methanol. Mass spectrometry data were acquired using an ESI source with switching between positive and negative ion modes, scanning range *m*/*z* 100–1500. The main instrument parameters were as follows: spray voltage, 3.5 kV; sheath gas pressure, 35 psi; auxiliary gas flow rate, 10 L/min; capillary temperature, 320 °C; S-lens RF voltage, 60; auxiliary heater temperature, 350 °C. MS/MS data were obtained using data-dependent acquisition (DDA) mode.

Raw data were processed using Compound Discoverer 3.3 software, with a mass tolerance of 5 ppm and a signal intensity threshold of 30%. Peak areas were normalized using the QC1 reference sample, and features with a coefficient of variation (CV) >30% among QC replicates were removed. Metabolite annotation was performed by matching accurate mass and fragmentation spectra against the mzCloud, mzVault, and MassList databases, and background ions were subtracted using procedural blank samples.

### 2.4. 16S rRNA High-Throughput Sequencing

Fresh fecal samples (5 g per bird) were collected from 10 randomly selected geese per group during peak laying and stored at −80 °C. Genomic DNA was extracted using a CTAB-based method and verified by agarose gel electrophoresis. The V3–V4 hypervariable region of the 16S rRNA gene was amplified using primers 341F (5′-CCTAYGGGRBGCASCAG-3′) and 806R (5′-GGACTACNNGGGTATCTAAT-3′) with Phusion^®^ High-Fidelity PCR Master Mix (NEB) (Ipswich, MA, USA) under the following conditions: 98 °C for 1 min; 30 cycles of 98 °C for 10 s, 50 °C for 30 s, and 72 °C for 30 s; final extension at 72 °C for 5 min. Purified amplicons were used to construct sequencing libraries (NEBNext^®^ Ultra DNA Library Prep Kit (Ipswich, MA, USA)) and sequenced on the Illumina platform (paired-end) at Shenzhen Weikezeng Technology Co., Ltd. (Shenzhen, China).

Bioinformatic processing was performed in QIIME2, with DADA2 used for quality filtering and ASV generation. Taxonomic assignment was performed against the GreenGenes database (trimmed to V3–V4, 99% similarity), and mitochondrial and chloroplast sequences were removed. Alpha diversity (observed species, Chao1, Shannon, Faith’s PD) and beta diversity (Bray–Curtis, unweighted/weighted UniFrac) were calculated and visualized by PCoA and NMDS. Differential abundance was assessed using ANCOM, one-way ANOVA, and Kruskal–Wallis tests. PLS-DA was performed using the R package mixOmics 4.2.3.

### 2.5. Statistical Analysis

Data were analyzed using SPSS 23.0. One-way ANOVA followed by Duncan’s multiple range test was used to compare differences between groups, while paired-sample t-tests were used for within-group comparisons. A *p*-value < 0.05 was considered statistically significant.

## 3. Results

### 3.1. Dietary Supplementation with Lactobacillus or Bacillus Improves Egg Production and Quality in Zhedong White Geese

The effects of probiotic supplementation on the production performance of Zhedong White geese are presented in Table 2. During the reproductive period, both the LAB and BAC groups exhibited increased total egg production compared to the Con group. Notably, the LAB group demonstrated a substantial increase in egg yield. While the fertilization rate was marginally higher in the LAB group (84.4%) than in the Con group (83.3%), the rates between the Con and BAC groups were comparable.

Regarding egg quality parameters, egg weight and albumen height did not differ significantly among the three groups. However, both probiotic groups showed significant improvements in key quality metrics. The Haugh unit, an indicator of protein quality, was significantly higher (*p* < 0.05) in the LAB (71.6 ± 0.70 HU) and BAC (69.0 ± 1.00 HU) groups compared to the Con group (66.6 ± 1.17 HU). Similarly, eggshell thickness was significantly greater (*p* < 0.05) in both the LAB (0.64 ± 0.03 mm) and BAC (0.61 ± 0.04 mm) groups than in the Con group (0.58 ± 0.02 mm).

Collectively, both probiotic treatments increased total egg output and yielded significant gains in Haugh unit and shell thickness relative to the Con group, while egg weight and albumen height remained statistically comparable across all three groups (Table 2).

### 3.2. PLS-DA Analysis of Serum Metabolites

Serum samples from 10 geese per group were profiled by untargeted metabolomics. A total of 629 and 481 features were detected in positive and negative ion modes, respectively. Pearson correlation coefficients among QC replicates exceeded 0.99 in both ionization modes, confirming instrument stability throughout data acquisition; features with a QC coefficient of variation above 30% were excluded before further analysis. Differential metabolites were identified on the basis of VIP > 1, FC > 2 or < 0.5, and *p* < 0.05.

**Figure 1:**
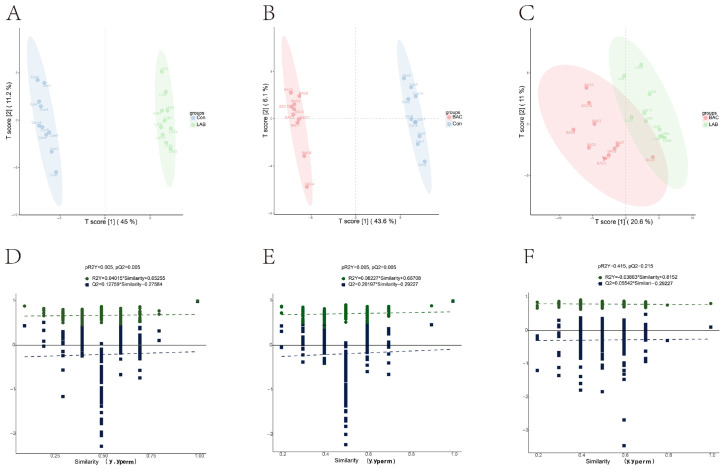
PLS-DA results and model validation. (**A**), PLS-DA scatter plot (LAB group vs. Con group); (**B**), PLS-DA scatter plot (BAC group vs. Con group); (**C**), PLS-DA scatter plot (LAB group vs. BAC group); (**D**), Validity diagram of model (LAB group vs. Con group); (**E**), Validity diagram of model (BAC group vs. Con group); (**F**), Validity diagram of model (LAB group vs. BAC group). Group abbreviations: Con, control group; LAB, group fed conventional diet supplemented with *Lactobacillus acidophilus*; BAC, group fed conventional diet supplemented with *Bacillus* spores.

PLS-DA of the curated feature matrix revealed distinct metabolic reprogramming in both probiotic groups relative to Con (Figure 1A,B), indicating that dietary supplementation with either strain drove reproducible and biologically meaningful shifts in circulating metabolite profiles. Direct comparison of the two probiotic groups, however, yielded no statistically significant separation (pR^2^Y = 0.415, pQ^2^ = 0.215; Figure 1C,F), with their profiles overlapping substantially, a pattern consistent with the comparable gains in egg quality observed for both treatments and suggestive of shared downstream regulatory targets. Model validity was established by permutation testing across all three comparisons: R^2^ exceeded Q^2^ in every case, and the Q^2^ regression line intersected the y-axis below zero, collectively excluding the possibility of overfitting (Figure 1D–F; pR^2^Y = pQ^2^ = 0.005 for LAB vs Con and BAC vs Con).

### 3.3. Screening of Common Differential Metabolites

**Figure 2:**
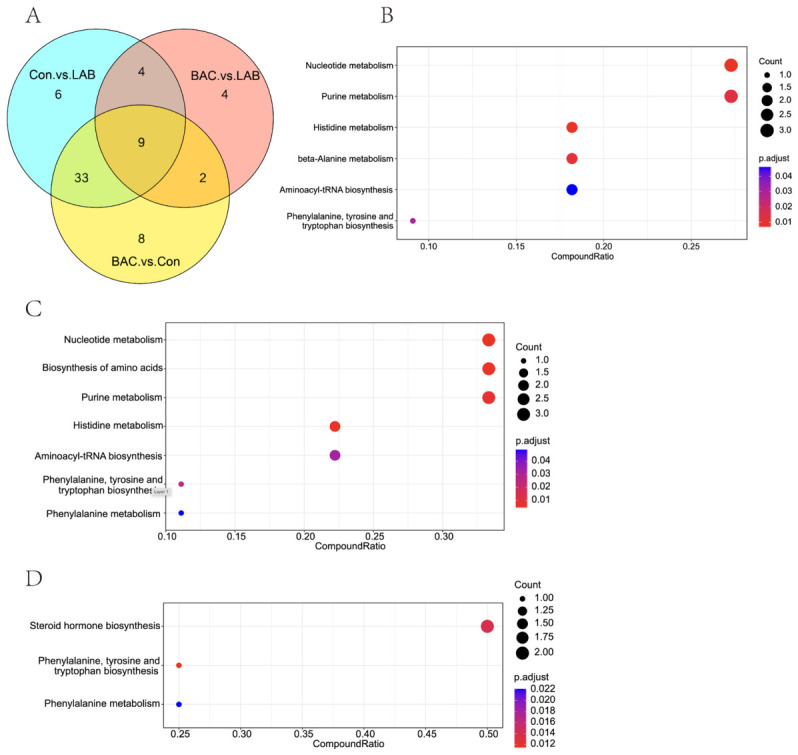
Common differential metabolites and KEGG enrichment analysis. (**A**), Analysis of different metabolites in each comparison group based on Venn diagram; (**B**), Differential metabolite enrichment bubble chart (LAB group vs. Con group); (**C**), Differential metabolite enrichment bubble chart (BAC group vs. Con group); (**D**), Differential metabolite enrichment bubble chart (LAB group vs. BAC group). Group abbreviations: Con, control group; LAB, group fed conventional diet supplemented with *Lactobacillus acidophilus*; BAC, group fed conventional diet supplemented with *Bacillus* spores.

By comparing the differentially expressed metabolites among the LAB, BAC, and Con groups, we identified nine common differential metabolites: 1,2-dihydroxyheptadec-16-yn-4-ylacetate, 2-methyl-2,3,4,5-tetrahydro-1,5-benzoxazepin-4-one, 3-[(4-chlorophenyl)thio]-1-phenylprop-2-en-1-one, D-Sphingosine, FRH, Hexanoylcarnitine, Indole, L-Phenylalanine, and LPE 22:6 (Figure 2A). These metabolites are associated with lipid metabolism, nervous system function, immune responses, and cellular energy management [12,13].

Notably, when we analyzed the changes in these metabolites across the groups, we found that all nine common metabolites exhibited identical trends of change in both the LAB and BAC groups when compared to the Con group. Specifically, 3-[(4-chlorophenyl)thio]-1-phenylprop-2-en-1-one, D-Sphingosine, Hexanoylcarnitine, L-Phenylalanine, and LPE 22:6 were upregulated, whereas 1,2-dihydroxyheptadec-16-yn-4-yl acetate, 2-methyl-2,3,4,5-tetrahydro-1,5-benzoxazepin-4-one, FRH, and Indole were downregulated (Table 3). This finding further indicates that feeding *Lactobacillus* and *Bacillus* alters the metabolic pathways of Zhedong White geese, and reinforces the conclusion that their modes of action share significant similarities.

### 3.4. KEGG Pathway Enrichment Analysis

To further elucidate the metabolic mechanisms through which *Lactobacillus* and *Bacillus* affect the production performance of Zhedong White geese, we performed a KEGG pathway enrichment analysis on the differential metabolites from the three groups. The differential metabolites between the LAB and Con groups were significantly enriched in a total of six metabolic pathways (Figure 2B): Nucleotide metabolism, Purine metabolism, Histidine metabolism, beta-Alanine metabolism, Aminoacyl-tRNA biosynthesis, and Phenylalanine, tyrosine and tryptophan biosynthesis. Compared to the Con group, the serum of geese in the LAB group showed significantly downregulated levels of Hypoxanthine, Xanthine, Guanine, and L-Histidine, while the levels of Carnosine and L-Phenylalanine were significantly upregulated (Table 4). KEGG enrichment analysis of the differential metabolites between the BAC and Con groups identified seven significant pathways (Figure 2C): Nucleotide metabolism, Purine metabolism, Histidine metabolism, Phenylalanine metabolism, Aminoacyl-tRNA biosynthesis, Phenylalanine, tyrosine and tryptophan biosynthesis, and Biosynthesis of amino acids. In comparison to the Con group, the BAC group exhibited significantly lower levels of Hypoxanthine, Xanthine, Guanine, and 1-Methylhistidine, and significantly higher levels of Carnosine, L-Phenylalanine, Methionine, and N-Acetylornithine (Table 4). Based on these results, it is evident that *Lactobacillus* and *Bacillus* co-modulate five common metabolic pathways in Zhedong White geese: Nucleotide metabolism, Purine metabolism, Histidine metabolism, Aminoacyl-tRNA biosynthesis, and Phenylalanine, tyrosine and tryptophan biosynthesis. Moreover, both probiotics led to the downregulation of Hypoxanthine, Xanthine, and Guanine, and the upregulation of Carnosine and L-Phenylalanine. In conjunction with their observed effects on production performance, we hypothesize that these common pathways may be pivotal for the improvement of egg quality in Zhedong White geese.

Furthermore, to identify pathways underlying the quantitative difference in egg output between groups, enrichment analysis of differential metabolites between LAB and BAC yielded three distinct pathways (Figure 2D): Phenylalanine, tyrosine and tryptophan biosynthesis; Phenylalanine metabolism; and Steroid hormone biosynthesis. Compared with the BAC group, the LAB group had significantly higher serum levels of Corticosterone and Tetrahydrocorticosterone, and significantly lower levels of L-Phenylalanine (Table 4). Notably, while L-Phenylalanine was elevated in both probiotic groups relative to Con, its concentration was significantly higher in BAC than in LAB (Table 4).

### 3.5. Lactobacillus and Bacillus Increased the Intestinal Microbial Diversity of Zhedong White Geese

**Figure 3:**
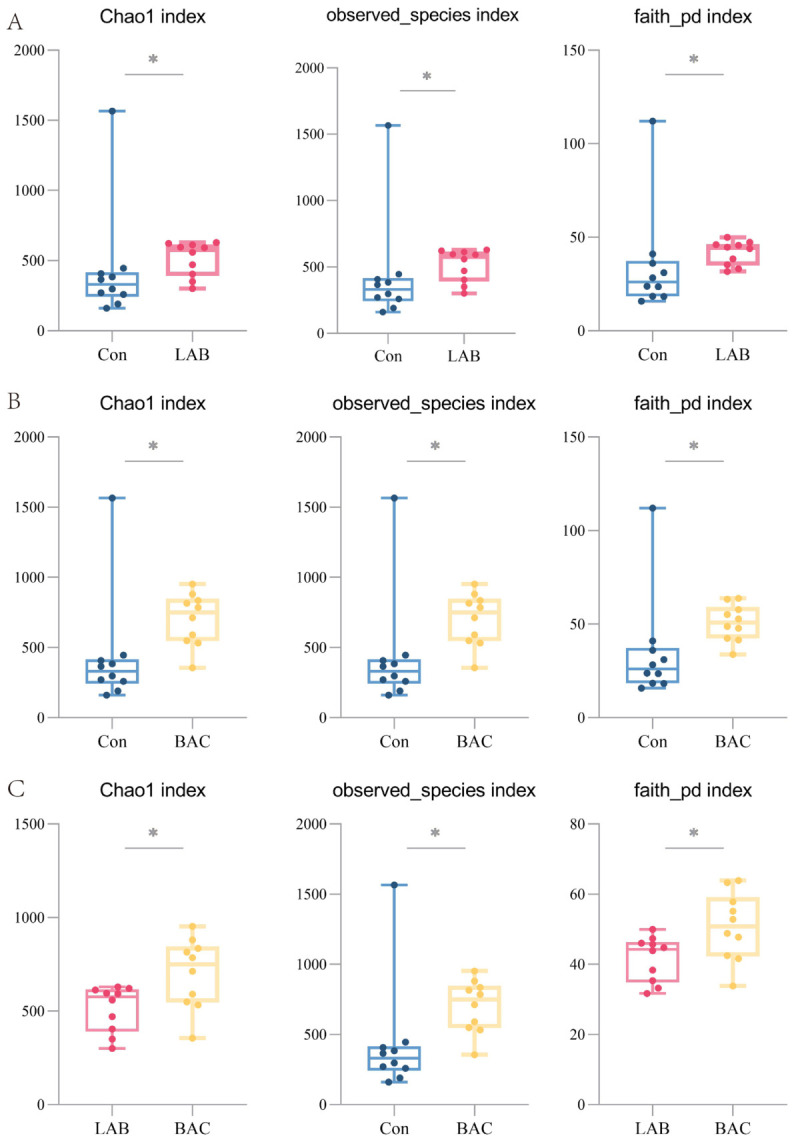
Effect of *Lactobacillus* and *Bacillus* on intestinal microbial diversity. (**A**), Comparison of the Chao1 index, observed species index, and Faith’s PD index of intestinal microorganisms in Zhedong white geese between the Con and LAB groups; (**B**), Comparison of the Chao1 index, observed species index, and Faith’s PD index of intestinal microorganisms in Zhedong white geese between the Con and BAC groups; (**C**), Comparison of the Chao1 index, observed species index, and Faith’s PD index of intestinal microorganisms in Zhedong white geese between the LAB and BAC groups. Group abbreviations: Con, control group; LAB, group fed conventional diet supplemented with *Lactobacillus acidophilus*; BAC, group fed conventional diet supplemented with *Bacillus* spores. * Represents *p* < 0.05.

Alpha diversity indices were used to analyze changes in the diversity of the gut microbiota. The Chao1 and observed_species indices reflect the richness of microbial species, while Faith’s PD index reflects the phylogenetic diversity of the microbial community. Compared to the Con group, the Chao1, observed_species, and Faith’s PD indices were all significantly higher in the gut microbiota of geese from both the LAB and BAC groups (Figure 3A,B). This indicates that dietary supplementation with either *Lactobacillus* or *Bacillus* can increase both the species richness and community diversity of the intestinal microbiota in Zhedong White geese. We further compared the changes in microbial diversity between the LAB and BAC groups and found that the Chao1, observed_species, and Faith’s PD indices were significantly higher in the BAC group than in the LAB group (Figure 3C). This suggests that, compared to *Lactobacillus*, *Bacillus* exerts a more pronounced effect on enhancing the overall diversity of the gut microbiota in Zhedong White geese.

### 3.6. Lactobacillus and Bacillus Modulated the Composition of the Intestinal Microbiota in Zhedong White Geese

**Figure 4:**
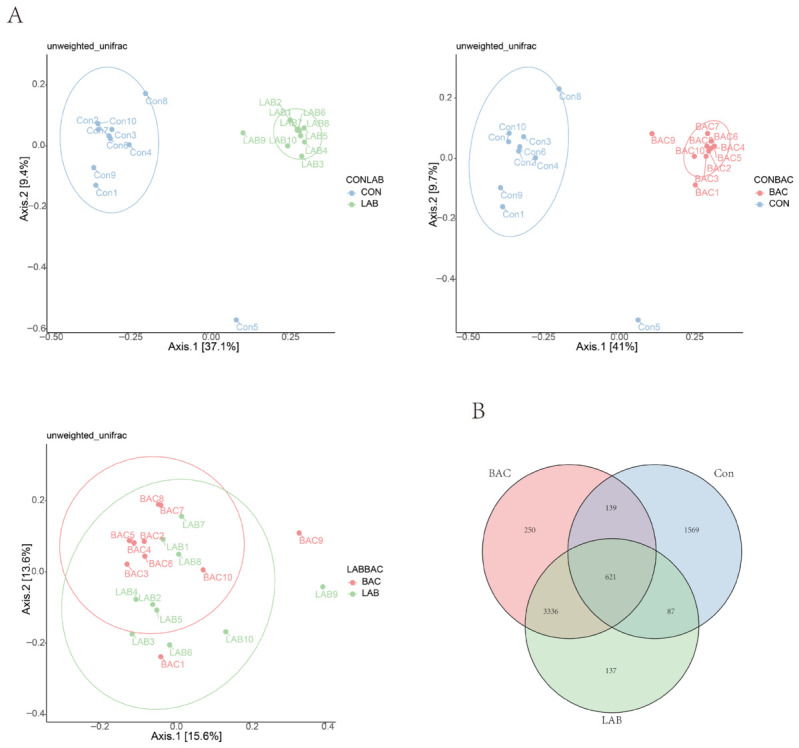
Effect of *Lactobacillus* and *Bacillus* on intestinal microbial diversity. (**A**), Chart of β-diversity results; (**B**), Venn’s diagram of species composition. Group abbreviations: Con, control group; LAB, group fed conventional diet supplemented with *Lactobacillus acidophilus*; BAC, group fed conventional diet supplemented with *Bacillus* spores. * Represents *p* < 0.05.

Beta diversity analysis is used to reflect the differences in microbial community structure between samples. We observed that the microbial communities of the Con group clustered separately from those of the BAC and LAB groups, while the LAB and BAC groups exhibited some overlap (Figure 4A,B). This finding is consistent with the metabolomics results, indicating that supplementation with both *Lactobacillus* and *Bacillus* altered the gut microbiota composition of Zhedong White geese, and that their modulatory effects share commonalities.

**Figure 5:**
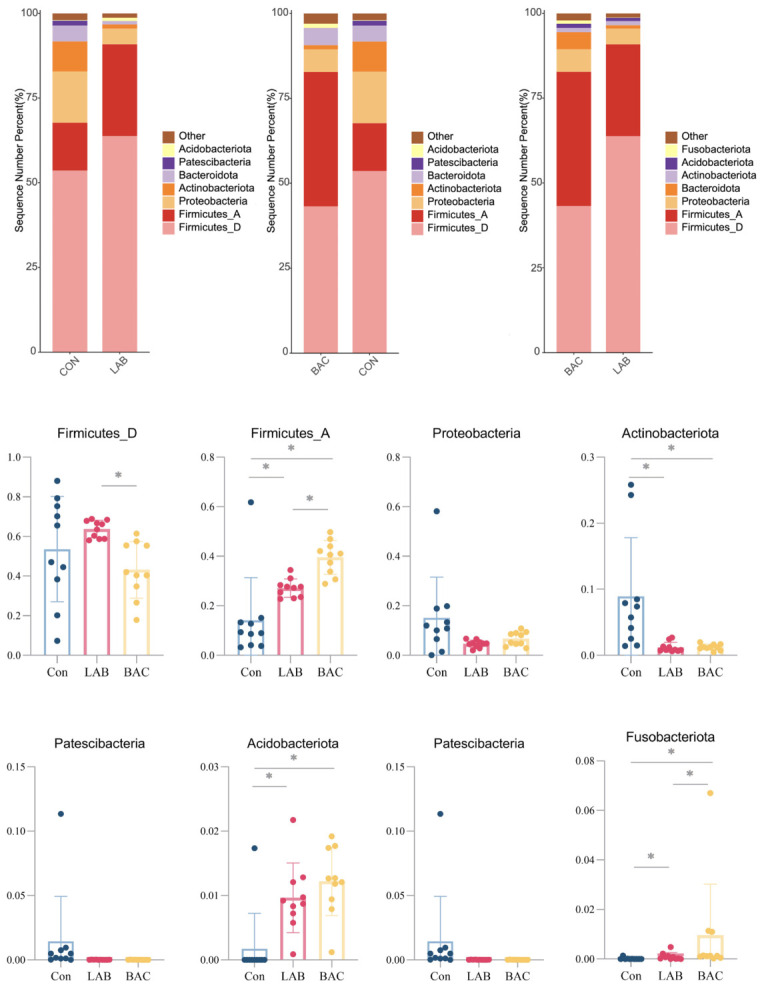
The Effects of *Lactobacillus* and *Bacillus* Species on Phylum-Level Species. Phylum-level species abundance and between-group comparisons of colony abundance. Group abbreviations: Con, control group; LAB, group fed conventional diet supplemented with *Lactobacillus acidophilus*; BAC, group fed conventional diet supplemented with *Bacillus* spores. * Represents *p* < 0.05.

At the phylum level, eight phyla with a relative abundance exceeding 1% were identified, with Firmicutes_D and Firmicutes_A as the dominant taxa in Zhedong White geese (Figure 5). Both probiotic groups exhibited higher Firmicutes abundance than the Con group, and supplementation significantly increased the relative abundance of Firmicutes_A, Acidobacteriota, and Fusobacteriota; the BAC group exerted a stronger effect on Firmicutes_A and Fusobacteriota than the LAB group. The abundance of Actinobacteriota was significantly reduced in both probiotic groups relative to the Con group.

**Figure 6:**
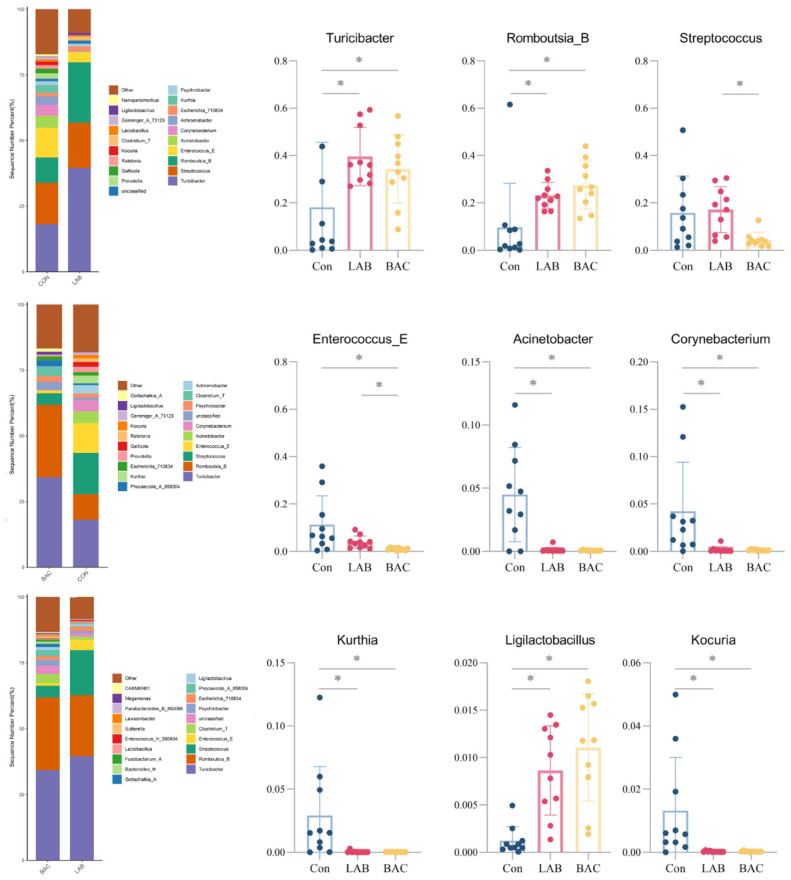
Effect of *Lactobacillus* and *Bacillus* on Genus-level species. Genus-level species abundance and between-group comparisons of colony abundance. Group abbreviations: Con, control group; LAB, group fed conventional diet supplemented with *Lactobacillus acidophilus*; BAC, group fed conventional diet supplemented with *Bacillus* spores. * Represents *p* < 0.05.

At the genus level, the Con group displayed a relatively uniform distribution of taxa, whereas both probiotic groups developed distinct dominant genera. Supplementation significantly increased the abundance of *Turicibacter, Romboutsia_B*, and *Ligilactobacillus*, and significantly decreased that of *Acinetobacter, Corynebacterium, Kurthia*, and *Kocuria* (Figure 6).

### 3.7. Correlation Analysis of Gut Microbiota and Metabolites

**Figure 7:**
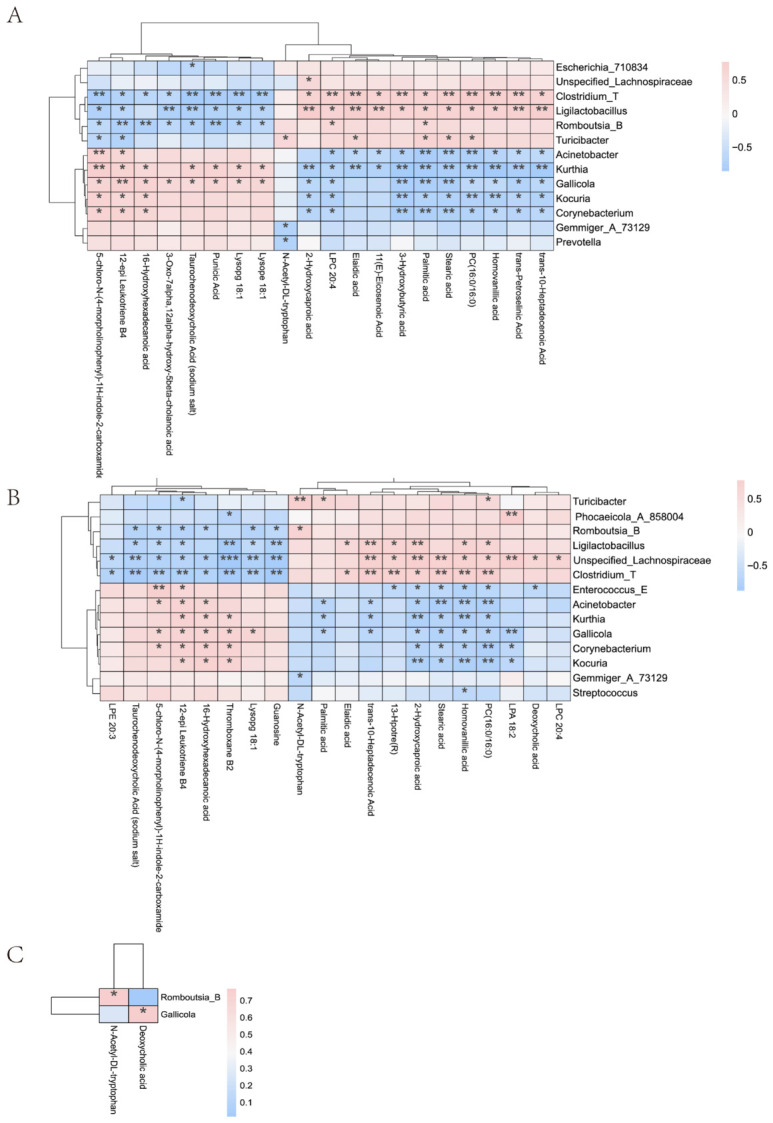
Correlation analysis between intestinal microbiota and metabolites. (**A**), Clustered heatmap of correlations between intestinal microbiota and metabolites in the Con and LAB groups; (**B**), Clustered heatmap of correlations between intestinal microbiota and metabolites in the Con and BAC groups; (**C**), Clustered heatmap of correlations between intestinal microbiota and metabolites in the LAB and BAC groups. Group abbreviations: Con, control group; LAB, group fed conventional diet supplemented with *Lactobacillus acidophilus*; BAC, group fed conventional diet supplemented with *Bacillus* spores. * Represents *p* < 0.05; ** Represents *p* < 0.01; *** Represents *p* < 0.001.

Correlation analysis between the gut microbiome and serum metabolome revealed consistent patterns across both probiotic groups. *Romboutsia_B*, *Turicibacter*, and *Ligilactobacillus* were positively correlated with fatty acid metabolites including 3-Hydroxybutyric acid, Palmitic acid, and Stearic acid, whereas *Acinetobacter, Corynebacterium, Kurthia, Kocuria,* and *Gallicola* were negatively correlated with these metabolites. Notably, *Romboutsia_B* showed a positive association with N-Acetyl-DL-tryptophan, and *Gallicola* was positively correlated with Deoxycholic acid (Figure 7A–C).

## 4. Discussion

Both *Lactobacillus* and *Bacillus* supplementation improved laying performance and egg quality in Zhedong White geese, yet their underlying mechanisms diverged considerably. *Lactobacillus* drove greater egg output principally through hormonal and metabolic signalling, while *Bacillus* acted more broadly on gut microbial composition and energy utilisation. The consistently higher total egg production in the LAB group reflects strain-level differences in reproductive engagement, whereas the comparable gains in shell thickness and Haugh unit between the two treatments point to shared influences on eggshell mineralisation and albumen deposition.

The total egg production in the LAB group was significantly higher than that in the BAC and Con groups, while there was little difference in egg quality between the two probiotic groups. This suggests that the key factors influencing egg production depend not only on nutritional supply but also on the regulation of reproductive signaling. Metabolomic analysis of the LAB and BAC groups further supported this view. *Lactobacillus* significantly activated the steroid hormone biosynthesis pathway, upregulating corticosterone and tetrahydrocorticosterone, while simultaneously enhancing aromatic amino acid metabolism. Although serum L-phenylalanine levels in both probiotic groups were higher than in the control group, the concentration in the LAB group was significantly lower than that in the BAC group. This suggests that *Lactobacillus* not only provides amino acid substrates but also promotes their conversion into catecholaminergic signaling molecules, thereby potentially modulating hypothalamic–pituitary–gonadal (HPG) axis activity to support the ovulatory process. *Lactobacillus* supplementation appeared to redirect phenylalanine more efficiently toward the catecholamine synthesis pathway. Phenylalanine is first hydroxylated to tyrosine, then to L-DOPA, and ultimately to dopamine and norepinephrine. These monoamine neuroactive substances stimulate the hypothalamus to release gonadotropin-releasing hormone (GnRH) and enhance the pulsatile secretion of luteinizing hormone (LH) from the pituitary [14]. Ovulation in poultry depends on a precise LH peak-triggering mechanism; even a modest increase in catecholaminergic tone can translate into a measurable increase in ovulation frequency. In contrast, the *Bacillus* group showed higher phenylalanine concentrations but lacked a corresponding hormonal pathway response, suggesting that accumulated amino acids were not fully utilized for reproductive signal transduction but were instead more involved in protein synthesis. This difference indicates that the improvement in reproductive performance depends not only on substrate supply but also on the dual regulation of metabolic flux and signaling pathways.

Meanwhile, the steroid hormone biosynthesis pathway was enriched only in the comparison between the LAB and BAC groups, and both serum corticosterone and its metabolite tetrahydrocorticosterone were significantly elevated in the LAB group. This finding further supported the aforementioned mechanism. It should be noted that corticosterone, as the most important stress hormone in poultry, inhibits the HPG axis and impairs reproductive function when sustained at chronically high levels [15,16]. Therefore, a more reasonable explanation is that corticosterone serves here as a detectable surrogate for increased metabolic flux through the upstream shared segment of the steroid hormone biosynthesis pathway. Specifically, cholesterol is converted to pregnenolone by side-chain cleavage enzyme; pregnenolone is then further converted to progesterone, subsequently entering two branching pathways: in the adrenal cortex, progesterone is converted to corticosterone by 11β-hydroxylase; in ovarian granulosa cells, progesterone is converted to estradiol by aromatase [17]. Activation of the upstream shared pathway is inevitably accompanied by a simultaneous increase in progesterone supply, and progesterone is the primary triggering hormone for the preovulatory LH surge in geese and other Anseriformes [18,19]. The fact that neither progesterone nor estradiol was detected as a statistically significant differential metabolite is more likely attributable to their low abundance and unfavorable ionization properties, rather than an absence of change in their concentrations. This hypothesis will be directly verified through targeted hormonal assays in future studies.

In terms of improving egg quality, both probiotics demonstrated positive effects, significantly increasing shell thickness and Haugh units, which indicates they not only boost egg numbers but also improve the structural integrity and protein quality of the eggs. The underlying mechanisms are likely associated with enhanced mineral absorption, increased activity of enzymes related to eggshell formation, and an improved systemic antioxidant capacity. This study detected a significant upregulation of Carnosine and aromatic amino acid metabolites. As an antioxidant dipeptide, Carnosine can scavenge reactive oxygen species, alleviate reproductive stress, and stabilize protein structures, thereby delaying the physiological decline in laying performance and extending the peak production period [20]. Furthermore, active aromatic amino acid metabolism not only provides substrates for protein synthesis but may also improve mineral transport by modulating neuro-endocrine signals, thus promoting calcium deposition in the eggshell [21,22]. Consistent with existing research, probiotics can improve eggshell strength by regulating the gut microbiota, enhancing calcium absorption efficiency, and promoting the production of short-chain fatty acids (SCFAs), which in turn reinforces intestinal epithelial function and acid–base homeostasis [23,24]. Concurrently, the activation of nucleotide metabolism (e.g., Purine metabolism) and Aminoacyl-tRNA biosynthesis pathways suggests that probiotics may increase protein translation efficiency, thereby improving albumen deposition and height. Our findings are consistent with reports in chickens and ducks on the egg quality-enhancing effects of probiotics, and by validating these effects in geese and uncovering the metabolic regulatory mechanisms, this study provides new evidence for understanding the molecular basis of improving egg quality in waterfowl.

Firmicutes_D and Firmicutes_A are the dominant phyla in the Zhedong White goose gut and play key roles in energy and nutrient metabolism. Firmicutes_A promotes SCFA production from dietary fibre and complex carbohydrates, thereby improving feed conversion efficiency [25], while Firmicutes_D contributes to protein and nitrogen utilisation, supporting muscle growth and productive performance [26]. Both probiotics significantly enriched Firmicutes_A, Acidobacteriota, and Fusobacteriota, with *Bacillus* exerting a particularly strong effect on Firmicutes_A and Fusobacteriota, consistent with its documented advantage in modulating energy metabolism. At the genus level, the probiotic-driven expansion of *Turicibacter* and *Ligilactobacillus* likely reinforces intestinal barrier integrity and mucosal immune defence, reducing inflammatory load across the flock [27]. The enrichment of *Romboutsia_B*, a taxon linked to protein and amino acid catabolism [28], may further support the elevated availability of substrates for egg biosynthesis. Conversely, the marked depletion of *Acinetobacter, Corynebacterium, Kurthia,* and *Kocuria*, which are recognised as potential pathogens, indicates a reduced intestinal pathogen burden and may relieve immune-mediated metabolic costs. Microbiome–metabolome correlation analysis extended these observations: the expanded beneficial genera correlated positively with fatty acid metabolites including 3-Hydroxybutyric acid and Palmitic acid, implicating them in lipid mobilisation and energy supply. The positive association between *Romboutsia_B* and N-Acetyl-DL-tryptophan further suggests upregulation of tryptophan catabolism feeding into the neuro-immune-endocrine axis, while the positive correlation between *Gallicola* and Deoxycholic acid implies that its depletion reduces secondary bile acid accumulation and alleviates hepatoenteric toxicity.

Interestingly, although the BAC group exhibited higher microbial diversity, stronger enrichment of energy metabolism pathways within Firmicutes, and broader activation of nitrogen utilization pathways, its egg production was lower than that of the LAB group. The fact that a greater degree of microbiome remodeling did not lead to a corresponding increase in egg production suggests that, while optimizing the gut ecosystem is essential, improvements at this level alone are insufficient to maximize egg production. In fact, the responsiveness of the endocrine system and the precise redirection of amino acid metabolic flux toward neuroendocrine signaling pathways are the key factors determining the upper limit of egg production performance.

This study combined laying performance records, gut microbiome profiles, and serum metabolome data to elucidate the distinct mechanisms by which different probiotic diets improve the egg production performance of Zhedong White geese. *Lactobacillus* primarily exerts its effects through the “aromatic amino acid–neuroendocrine–steroid hormone” axis. Accelerated catabolism of phenylalanine enhances catecholamine signaling at the hypothalamic level, while the simultaneous upregulation of steroid hormone biosynthesis pathways may promote ovarian progesterone supply, together supporting completion of the ovulatory cascade. Bacillus, on the other hand, enhances the overall production potential of the gut ecosystem by enriching energy-harvesting and nitrogen-metabolizing microbial communities. The fundamental mechanistic differences between these two probiotics provide practical evidence for precise selection of microbial strains in antibiotic-free goose farming and offer new insights into how the interactions among the gut microbiome, metabolites, and the host endocrine axis shape avian reproductive performance.

## 5. Limitations

However, this study has several limitations: (1) The experimental period covered only a single reproductive cycle and lacks long-term validation. (2) The study did not evaluate dose-gradient effects or the effects of combined probiotic supplementation. (3) The metabolomics and microbiome analyses are correlational in nature; causal relationships need to be verified through further studies, such as transcriptomics or in vivo intervention experiments. In the future, we will aim to further explore the mechanisms of these probiotics at different production stages and optimize their supplementation strategies. (4) Although the metabolomics results of this study identified corticosterone and tetrahydrocorticosterone as differential metabolites in the steroid hormone biosynthesis pathway, quantification of low-abundance reproductive steroids sharing the same biosynthetic upstream remains insufficient. 

## Figures and Tables

**Table 1:** Ingredient composition and nutrient levels of the basal diet (air-dry basis).

Ingredient	Content (%)	Nutrient Level	Value
Rice	13.00	ME (MJ/kg) ^2^	11.98
Barley	12.00	CP (%)	15.54
Wheat	14.00	Ca (%)	0.26
Rice bran	5.00	P (%)	0.40
Wheat bran	5.00		
Alfalfa meal	12.00		
Corn	30.00		
Soybean meal	8.00		
Premix ^1^	1.00		
Total	100.00		

^1^ Premix supplied per kg of diet: Cu 2 g; Fe 4 g; Se 0.25 g; Zn 5 g; vitamin E 12 g; vitamin C 60 g; vitamin A 5 g. ^2^ ME = metabolizable energy (calculated value); all other nutrient levels are measured values. CP = crude protein; Ca = calcium; P = phosphorus.

**Table 2:** The effects of *Lactobacillus* and *Bacillus* on the production performance of Zhedong white geese.

Group	Total Egg Production	Fertilization Rate (%)	Egg Weight (g)	Shell Thickness (mm)	Albumen Height (mm)	Haugh Unit (HU)
Con	41,538	83.3	163.3 ± 11.3	0.58 ± 0.02	8.3 ± 0.38	66.6 ± 1.17
LAB	45,849	84.4	163.6 ± 12.2	0.64 ± 0.03 *	8.7 ± 0.47	71.6 ± 0.70 *
BAC	42,272	83.4	163.8 ± 13.2	0.61 ± 0.04 *	8.5 ± 0.47	69.0 ± 1.00 *

Values are expressed as mean ± SD. * *p* < 0.05 vs. Con group. Con, control group fed basal diet without supplementation; LAB, group fed basal diet supplemented with *Lactobacillus acidophilus*; BAC, group fed basal diet supplemented with *Bacillus subtilis*.

**Table 3:** Changes in differential metabolites among groups.

Differential Metabolites	LAB vs. Con	BAC vs. Con	LAB vs. BAC
1,2-dihydroxyheptadec-16-yn-4-yl acetate	DOWN	DOWN	UP
2-methyl-2,3,4,5-tetrahydro-1,5-benzoxazepin-4-one	DOWN	DOWN	DOWN
3-[(4-chlorophenyl)thio]-1-phenylprop-2-en-1-one	UP	UP	UP
D-Sphingosine	UP	UP	DOWN
FRH	DOWN	DOWN	DOWN
Hexanoylcarnitine	UP	UP	UP
Indole	DOWN	DOWN	DOWN
L-Phenylalanine	UP	UP	DOWN
LPE 22:6	UP	UP	UP

Con, control group fed basal diet without supplementation; LAB, group fed basal diet supplemented with *Lactobacillus acidophilus*; BAC, group fed basal diet supplemented with *Bacillus subtilis*.

**Table 4:** KEGG enriched pathways and differential Metabolites.

Group	Pathway	Upregulated Metabolites	Downregulated Metabolites
LAB vs. Con	Nucleotide metabolism		Hypoxanthine, Xanthine, Guanine
	Purine metabolism		Hypoxanthine, Xanthine, Guanine
	Histidine metabolism	Carnosine	L-Histidine
	beta-Alanine metabolism	Carnosine	L-Histidine
	Aminoacyl-tRNA biosynthesis	L-Phenylalanine	L-Histidine
	Phenylalanine, tyrosine and tryptophan biosynthesis	L-Phenylalanine	
BAC vs. Con	Nucleotide metabolism		Hypoxanthine, Xanthine, Guanine
	Purine metabolism		Hypoxanthine, Xanthine, Guanine
	Histidine metabolism	Carnosine	1-Methylhistidine
	Phenylalanine metabolism	L-Phenylalanine	
	Aminoacyl-tRNA biosynthesis	L-Phenylalanine, Methionine	
	Biosynthesis of amino acids	L-Phenylalanine, Methionine, N-Acetylornithine	
	Phenylalanine, tyrosine and tryptophan biosynthesis	L-Phenylalanine	
LAB vs. BAC	Phenylalanine, tyrosine and tryptophan biosynthesis		L-Phenylalanine
	Phenylalanine metabolism		L-Phenylalanine
	Steroid hormone biosynthesis	Corticosterone, Tetrahydrocorticosterone	

## Data Availability

The original contributions presented in this study are included in the article. Further inquiries can be directed to the corresponding authors.

## References

[1] Yogeswari M.S., Selamat J., Jambari N.N., Khatib A., Mohd Amin M.H., Murugesu S. (2024). Metabolomics for quality assessment of poultry meat and eggs. Food Qual. Saf..

[2] Li W., Zhao M., Zhang L., Sun G., Zhao H., Zhang G., Ji R., Wang J., Li X., Chen G. (2025). Impact of low-protein diet on geese growth: Early low-protein diets with amino acid supplementation improve nitrogen utilization and maintain growth performance in meat geese. Front. Anim. Sci..

[3] Bava R., Castagna F., Lupia C., Poerio G., Liguori G., Lombardi R., Naturale M.D., Mercuri C., Bulotta R.M., Britti D. (2024). Antimicrobial Resistance in Livestock: A Serious Threat to Public Health. Antibiotics.

[4] Magnusson U. (2025). Medically rational use of antibiotics to reduce resistance and support effective animal production in low-and middle-income countries. Rev. D’élevage Médecine Vétérinaire Pays Trop..

[5] Enshaie E., Nigam S., Patel S., Rai V. (2025). Livestock Antibiotics Use and Antimicrobial Resistance. Antibiotics.

[6] Leistikow K.R., Beattie R.E., Hristova K.R. (2022). Probiotics beyond the farm: Benefits, costs, and considerations of using antibiotic alternatives in livestock. Front. Antibiot..

[7] Dec M., Puchalski A., Urban-Chmiel R., Wernicki A. (2014). Screening of Lactobacillus strains of domestic goose origin against bacterial poultry pathogens for use as probiotics. Poult. Sci..

[8] Gresse R., Cappellozza B.I., Macheboeuf D., Torrent A., Danon J., Capern L., Sandvang D., Niderkorn V., Copani G., Forano E. (2025). In Vitro Investigation of the Effects of Bacillus subtilis-810B and Bacillus licheniformis-809A on the Rumen Fermentation and Microbiota. Animals.

[9] Tajudeen H., Ha S.H., Hosseindoust A., Mun J.Y., Park S., Park S., Choi P., Hermes R.G., Taechavasonyoo A., Rodriguez R. (2024). Effect of dietary inclusion of Bacillus-based probiotics on performance, egg quality, and the faecal microbiota of laying hen. Anim. Biosci..

[10] Dong R., Liu H., Zhang H., Wu F., Xiu H., Chen S., Yin X., Zhou X. (2025). Effects of Bacillus subtilis ZY1 on production performance, egg quality, serum parameters and intestinal health in laying hens. Poult. Sci..

[11] Chen J., Dong B., Fan X., Ge J., Zhao M., Liu L., Gong D., Wang J., Geng T. (2025). Supplemented *Clostridium butyricum* and *Bacillus subtilis* affects the physiology and production performance of Yangzhou geese. Ital. J. Anim. Sci..

[12] Xiang F., Zhang Z., Xie J., Xiong S., Yang C., Liao D., Xia B., Lin L. (2025). Comprehensive review of the expanding roles of the carnitine pool in metabolic physiology: Beyond fatty acid oxidation. J. Transl. Med..

[13] Kumar P., Lee J.H., Lee J. (2021). Diverse roles of microbial indole compounds in eukaryotic systems. Biol. Rev. Camb. Philos. Soc..

[14] Liu X., Chen X., Wang C., Song J., Xu J., Gao Z., Huang Y., Suo H. (2024). Mechanisms of probiotic modulation of ovarian sex hormone production and metabolism: A review. Food Funct..

[15] Henriksen R., Groothuis T.G., Rettenbacher S. (2011). Elevated plasma corticosterone decreases yolk testosterone and progesterone in chickens: Linking maternal stress and hormone-mediated maternal effects. PLoS ONE.

[16] Ahmed A.A., Ma W., Ni Y., Wang S., Zhao R. (2014). Corticosterone in ovo modifies aggressive behaviors and reproductive performances through alterations of the hypothalamic-pituitary-gonadal axis in the chicken. Anim. Reprod. Sci..

[17] Payne A.H., Hales D.B. (2004). Overview of steroidogenic enzymes in the pathway from cholesterol to active steroid hormones. Endocr. Rev..

[18] Brady K., Liu H.C., Hicks J., Long J.A., Porter T.E. (2023). Global gene expression analysis of the turkey hen hypothalamo-pituitary-gonadal axis during the preovulatory hormonal surge. Poult. Sci..

[19] Qin Q., Sun A., Guo R., Lei M., Ying S., Shi Z. (2013). The characteristics of oviposition and hormonal and gene regulation of ovarian follicle development in Magang geese. Reprod. Biol. Endocrinol..

[20] Boldyrev A.A., Aldini G., Derave W. (2013). Physiology and pathophysiology of carnosine. Physiol. Rev..

[21] Han Q., Phillips R.S., Li J. (2019). Editorial: Aromatic Amino Acid Metabolism. Front. Mol. Biosci..

[22] Lu Y., Xu H., Hu Z., Li D., Rustempasic A., Zhou Y., Deng Q., Pu J., Zhao X., Zhang Y. (2025). Probiotics improve eggshell quality via regulating microbial composition in the uterine and cecum. Poult. Sci..

[23] Wang Y., Zhang C., Chen X., Zheng A., Liu G., Ren Y., Chen Z. (2024). Dietary supplementation of compound probiotics to improve performance, egg quality, biochemical parameters and intestinal morphology of laying hens. Front. Vet. Sci..

[24] Rezaeipour M., Afsharmanesh M., Khajeh Bami M. (2022). Evaluation of the effect of short-chain organic acids and probiotics on production performance, egg white quality, and fecal microbiota of laying hens. Comp. Clin. Pathol..

[25] Xu Q., Yuan X., Gu T., Li Y., Dai W., Shen X., Song Y., Zhang Y., Zhao W., Chang G. (2017). Comparative characterization of bacterial communities in geese fed all-grass or high-grain diets. PLoS ONE.

[26] Wen K., Liu L., Zhao M., Geng T., Gong D. (2022). The Changes in Microbiotic Composition of Different Intestinal Tracts and the Effects of Supplemented Lactobacillus During the Formation of Goose Fatty Liver. Front. Microbiol..

[27] Huang Y., Xu L., He H., Peng L., Liao Q., Wan K., Qin S., Cao L., Zhang J. (2024). Effects of rosemary extract and its residue on production, immune performance, and gut microbiota in geese. Front. Microbiol..

[28] Guo B., Li D., Zhou B., Jiang Y., Bai H., Zhang Y., Xu Q., Zhao W., Chen G. (2019). Comparative characterization of bacterial communities in geese consuming of different proportions of ryegrass. PLoS ONE.

